# Are We Over-Drugging Ourselves ?

**Published:** 1923-07

**Authors:** 


### ARE WE OVER-DRUGGING OURSELVES?

"NINE out of every ten bottles of medicine prescribed are entirely useless to the persons taking them." That is an arresting statement and one of the utmost gravity. But there is worse to follow. "And are known to be useless by the doctors who prescribe them." In a letter recently published by us, a consultant who practises in an industrial area, drew attention to the "bottle-of-medicine habit." In an earlier interview with our Special Commissioner some very pointed observations on the same subject were made by Professor Wynne, of Sheffield University, the Medical Officer of that city. In particular, he said that it was a matter for regret that the National Health Insurance scheme had not educated the insured person away from the fetish of the eight-ounce bottle of medicine. And now we have the serious statement which we have quoted at the beginning of this article. It is no idle tilt at the panel system, or what is sometimes thought to be panel medicine, by some irresponsible person in a hastily-written letter to his daily paper. It is the considered expression of the view of a general practitioner with one of the largest working-class practices in he country, Dr. Harry Roberts, embodied in his recently-published book "A National Health Policy," after he has had the opportunity of seeing how it looks in cold print in the proof-sheets. It applies equally to private and panel practice, as Dr. Roberts makes clear in another passage, in which he repeats the statement in substance, but softens the blow just a little by the qualification that nine-tenths of all the bottles of medicine are mere placebos, having none but a psychological effect on their recipients.

The doctor's calling is a high one, and he is engaged against powerful opposing forces in a struggle for the existence of his patients. But doctors have to live like lesser mortals, and are engaged on their own account in the fierce struggle for existence. This struggle, unlike the other, is not with outside forces, but with one another, and if, indeed, it leads to a perpetual pandering to the ignorant prejudices of their patients, it is, to use Dr. Roberts' own expression, indistinguishable from prostitution. We have all been brought up in the belief that a visit from the doctor must inevitably be followed by one or more bottles of mixture, and some of us have anxiously watched the hands of the clock "every four hours," lest some evil befall us by swallowing our dose at ten minutes past eight instead of at eight o'clock when it fell due. First the bedside manner, then the bottle. And now, are we to be rudely disillusioned? If so, let the awakening come by an announcement, in no haltin- or tentative tones, from those who can speak for doctors as a body. Human nature being what it is, we shall not expect Dr. Roberts or any other individual practitioner to practice what he preaches if it means that all his patients will go to the other doctor round the corner. It is too much to expect. Let us have propaganda, that prostitution may hide its unclean head.

Nine out of every ten bottles! It is a challenge to the whole medical profession from one of their number, to say nothing of the chemists and the bottle-manufacturers. The bottle in its many forms stands in our journey from the cradle to the grave for so much to all of us that the *obiter dictum* comes as a rude shock. We draw the attention of our hospitals and our doctors to the challenging statement which has been made. What they have to say on the subject will be of absorbing interest to the layman who studies public health questions and to whom in our columns we especially make appeal.

